# Research on a Fractal Dimension Calculation Method for a Nano-Polymer Microspheres Dispersed System

**DOI:** 10.3389/fchem.2021.732797

**Published:** 2021-09-20

**Authors:** Wenyue Zhao, Jinming Yan, Ganggang Hou, Pengxiang Diwu, Tongjing Liu, Jirui Hou, Ruolin Li

**Affiliations:** ^1^ The Unconventional Oil and Gas Institute, China University of Petroleum, Beijing, China; ^2^ Institute of Artificial Intelligence, China University of Petroleum, Beijing, China; ^3^ College of Science, China University of Petroleum, Beijing, China

**Keywords:** fractal dimension, polymer microspheres, box counting model, cumulative probability distribution, hydration swelling

## Abstract

Polymer microspheres (PMs) are a kind of self-similar volume expansion particle, and their fractal dimension varies with hydration swelling. However, there is no unique fractal dimension calculation method for their characteristics. A new model is established in this paper, which is particular to calculate the fractal dimension of PMs. We carried out swelling hydration experiments and scanning electron microscope (SEM) experiments to verify the new model. The new model and the box-counting model were used to calculate the fractal dimensions of PMs based on the hydration experiment results. Then, a comparison of the calculation results of the two methods was used to verify the validity of the model. Finally, according to the new model calculation results, the fractal dimension characteristics of PMs were analyzed. The research results indicate that the new model successfully correlates the cumulative probability of the PMs dispersed system with the fractal dimension and makes fractal dimension calculation of PMs more accurate and convenient. Based on the experiment results, the new model was used to calculate the fractal dimension of PMs and the box-counting model, and its findings were all 2.638 at initial state hydration and 2.739 and 2.741 at hydration time as of day 1. This result verifies the correctness of the new model. According to the hydration swelling experiments and the new model calculation results, the fractal dimension is linear correlated to the average particle size of PMs and the standard deviation average particle size. This means the fractal dimension of PMs represents the space occupancy ability and space occupancy effectiveness.

## Introduction

Polymer microspheres (PMs) are spherical polymer composite materials with diameters ranging from nanometers to micrometers, high specific surface area, high reactivity, and other unique physical, chemical, biological properties (Wang et al., 2019; Wang et al., 2018). PMs have a wide range of applications in the pharmaceutical industry, textile industry, energy industry, and other fields (Shen et al., 2016; Zhao et al., 2018; Liu et al., 2018; Liao et al., 2016). PMs have therefore become one of the most eye-catching research directions in functional polymer materials and related fields in recent years (Jia et al., 2018).

Among its many application fields, oil development is one of the most important application fields (Kawaguchi, 2000; Raffa et al., 2016; Jia et al., 2019) because it has many excellent properties, such as stability, rheology, and self-similarity (Kang et al., 2015; Yang et al., 2017). Among them, oilfield researchers are most concerned with properties such as the PMs particle size and its distribution. In the oilfield, researchers apply the PMs to plug the porous media in the reservoir to expand the swept volume of injected water and improve oil recovery, which is called in-depth conformance control technology (Liu et al., 2016). However, the effect of in-depth conformance control technology with PMs depends on whether the particle size of PMs matches the throat’s diameter (Pu et al., 2018). At present, the selection method of PMs is based mainly on the matching coefficient that comes from the ratio of the particle size with the throat diameter (Yang et al., 2017). This method considered only the average particle size of the PMs, which overlooks the particle size distribution of the PMs and results in some PMs not matching the throats. Therefore, a new method is needed to characterize the microsphere dispersion system and describe the particle distribution and particle size simultaneously. At this time, the self-similar characteristics of PMs have attracted the attention of researchers. For a disordered polymer system microspheres dispersed solution, the microstructure can be named self-similar portions present in a matrix called fractals (Sayan et al., 2018). That is because the system that satisfies the self-similar theory can study its particle size distribution rule according to fractal theory (Hu et al., 2006).

In 1977, Mandelbrot proposed a fractal dimension theory that described the self-similarity, nature fracture, and irregular structures (Mandelbrot, 1982; Katz et al., 1985; Xia et al., 2018). In the past few decades, fractal modeling and its associated concepts have been extensively applied to many areas such as mathematics, physics (Poushali et al., 2019), chemistry (Sayan et al., 2015), and Earth science (Yu et al., 2001; Yu et al., 2002). To date, there are six ways to define fractal dimension: similarity dimension, capacity dimension, box dimension, formation dimension, correlation dimension, and generalized dimension. There are also five ways to measure the dimensions: changing the degree of megascopic, applying correlation of measurements, utilizing correlative functions, applying distribution functions, and analyzing the spectrum (Chen, 1999; Sun, 2004). In these methods, the box dimension method is the most commonly used. That is because its calculation method is simple and easy to understand. However, for the PMs, the calculation results are greatly affected by the observation position. That is because the particle size distribution of PMs in the same sample is nonuniform. A particular new method needs to be established to calculate the fractal dimension of PMs dispersion system to solve this problem.

In this paper, to calculate PMs’ fractal dimension more accurately and conveniently, a new model was established, which successfully correlates the cumulative probability of the PMs dispersed system with the fractal dimension based on the self-similarity theory. We carried out swelling hydration experiments and scanning electron microscope (SEM) experiments to verify the new model and obtained the data needed to calculate the fractal dimension of PMs. Then, the new model and the conventional calculation model (box counting model) were used to calculate the fractal dimension of PMs at different hydration times. The calculation results verify the correctness of the new model. Finally, according to the calculation results of the new model, the fractal dimension characteristics of PMs were analyzed.

## Polymer Microspheres Fractal Dimension Calculation Model

### Cumulative Probability Model Building

There are many popular kinds of fractal dimensions, as stated by definition. According to the fractal geometry theory, for the statistically self-similar system of the PMs, an essential relation of fractal scaling law between microsphere accumulated number *N(>λ)* and microsphere size *λ* can be presented as following (Zhang et al., 2004).

$$N\left(>\lambda \right)\infty \lambda^{-D}$$ (1)

Where *N(>λ)* is the number of particles corresponding to the size and the particle size, and D is the fractal dimension of PMs dispersed system.

*N*_*t*_ is the total number of microspheres participating in the statistics in the microsphere system, *λ*_min_ is the minimum diameter of the microsphere, and *f* (*λ*_min_) is the probability corresponding to the minimum diameter. The limitation of *N(>λ)* can be presented as the following equation as:

$$\underset{\lambda \to \lambda_{\min}}{\lim}N\left(>\lambda \right)=N_{\text{t}}\left(1-f\left(\lambda_{\min}\right)\right)=K\lambda_{\min}^{-D}$$ (2)

Therefore, the slope *K* can be expressed as:

$$K=N_{\text{t}}\left(1-f\left(\lambda_{\min}\right)\right)\lambda_{\min}^{D}$$ (3)

Where *K* is the equality, Eq. 1 can be expressed as:

$$\text{N}\left(>\text{λ}\right)={\text{N}}_{\text{t}}\left(1-f\left(\lambda_{\min}\right)\right){\left(\frac{\lambda_{\min}}{\lambda }\right)}^{D}$$ (4)

As in the probability theory, the cumulative probability density function *P(λ)* should be following:

$$P\left(\lambda \right)=1-\left(1-f\left(\lambda_{\min}\right)\right){\left(\frac{\lambda_{\min}}{\lambda }\right)}^{D}$$ (5)

The fractal dimension can be calculated using the curve of Eq. 5 to nonlinearly fit the cumulative probability density of PMs that correspond to the size. Standard methods can measure the particle size distribution data, including dynamic light scattering, SEM, and transmission electron microscope (Han, 2007).

From Eq. 4, the number of PMs radius lying between *λ* to *λ+dλ* following as following:

$$-dN=DN_{t}\left(1-f\left(\lambda_{\min}\right)\right)\lambda_{\min}^{D}\lambda^{-\left(D+1\right)}d\lambda$$ (6)

**FIGURE 1:**
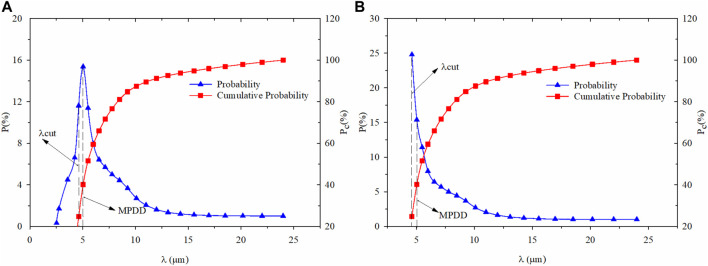
The microsphere size distribution is based on dynamic scattering. **(A)** Initial state microsphere size distribution; **(B)** Microsphere size distribution after number accumulating at cut value.

The negative sign in Eq. 6 implies that the microsphere decreases as the microsphere size increase and -dN>0. Therefore, the full range of microsphere size should follow a monotonically decreasing distribution, as shown in Figure 1. In contrast, the actual size distribution of PMs usually exhibits left-skewed distribution. The peak value of probability is located in a smaller microsphere diameter and is always more significant than the median and mean number presented in Figure 1. Thus, in calculating the fractal dimension with the microsphere cumulative probability distribution data, the difference between a theoretical fractal distribution and a negatively skewed distribution will reduce data fitting accuracy. The difference will cause the calculation error of fractal dimension because only the self-similar fractal interval of the PMs follows the fractal scaling rule, not all the entire range.

We need to find the microsphere diameter corresponding to the microsphere’s maximum numbers to obtain accurate fractal dimension values. We define it as a maximum probability density diameter (MPDD) for the probability distribution of particle size larger than MPDD, obeying a monotonical decreasing rule.

The accumulated number, corresponding to a microsphere size that is less than the MPDD, can be considered integration to replace the original minimum diameter *λ*_min_ concerning numbers from the minimum *λ*_*cut*_. The cut value *λ*_*cut*_ can be directly obtained from probability density, which is the previous radius value of MPDD. Figure 1 shows the microsphere size distribution obtained from the dynamic lighting scattering. After that, it processes the data with the formers method and compares the probability density of PMs from the minimum diameter to *λ*_*cut*_ (Figure 1B) with the original probability density distribution (Figure 1A). The full range of accumulated microsphere size distribution following the fractal scaling rule is concluded, which can be used to calculate the accurate fractal dimension values.

Replace *λ*_min_ with *λ*_*cut*_, the relationship between cumulative probability and fractal dimension can be expressed as:

$$P\left(\lambda \right)=1-\left(1-f\left(\lambda_{cut}\right)\right){\left(\frac{\lambda_{cut}}{\lambda }\right)}^{D}$$ (7)

### Box Counting Model Analysis

The box counting model is one of the commonly used methods to calculate the fractal dimension of the PMs dispersion system. To validate the cumulative probability model, we also use the box-counting method to calculate the fractal dimension of the same PMs.

The fractal dimension *D* of the PMs dispersion system can be calculated using the box counting method based on SEM analysis and light microscope experiment results. The cross-section involved is discretized using square boxes of size *r*, then the number *N* of boxes required to cover the microsphere ultimately is counted. The data slope value can determine the fractal dimension of PMs on the logarithmic plot. Theoretically, if the image is fractal, *N* and *r* follow as:

$$N=r^{-D_{b2}}$$ (8)

Where *D*_*b2*_ is the 2D fractal dimension of the box-counting method, and the 3D polymer microsphere, Eq. 9 can calculate the fractal dimension (Hu, 2006);

$$D_{b3}=D_{b2}+1$$ (9)

## Cumulative Probability Model Validation

### Experimental Section

#### Materials

Bohai Oilfield provides the PMs used in the experiment. Its main component was polymer and synthesized by reverse phase emulsion and reverse-phase suspension. The physical and chemical properties of this PMs indicated that it was an environmentally friendly PMs. It was non-toxic and non-corrosive and was kept in white oil with an acceptable solid content of 20%. The anhydrous ethanol (CH_3_CH_2_OH, purity above 99.5%) was used as the dispersion medium to make a uniform dispersion solution of PMs in the initial state. The simulated formation water was used as the solvent to study the hydration swelling properties of PMs. Bohai Oilfield provides its ion composition, and the total salinity was 9,500 mg/L. The SEM experiment is carried out by using liquid nitrogen to freeze the PMs.

#### Experimental Setup

We divided the experiment into two parts. One part of the experiment studied the hydration swelling properties of PMs. The main equipment used in this experiment was Ultrasonic Instrument (produced by Tianjin Autoscience Instrument Co., Ltd., China.) and Nanoparticle Size Analyzer (produced by Beckman Coulter, USA). Ultrasonic Instrument was primarily used to disperse the PMs solution. Its ultrasonic frequency and rated powers were 40 kHz and 120 W, respectively. Nanoparticle Size Analyzer was mainly used to measure the diameter of PMs and its measurement range is 0.6 nm–7 μm. Other equipment, such as thermostats, quartz cuvettes (10 ml), and electromagnetic stirrers, were also used in the experiment. The other experiment was to obtain the morphology and particle size distribution of PMs. The main equipment used in this experiment was a light microscope (BX-41) manufactured by Olympus Corporation in Japan and a scanning electron microscope (Quanta 200F) manufactured by FEI in the United States.

#### Experimental Procedure

##### Hydration Swelling Experiment.

The main purpose of the hydration swelling experiment is to measure the particle size distribution of the PMs dispersion system. The experiment was carried out at 65 C, and the experiment data was measured at 0, 1, 3, 5,10, and 30 days. The experimental procedures of hydration swelling properties are summarized as follows:

1) Take 100 ml of anhydrous ethanol into a beaker and add an appropriate amount of PMs. Stir it to get the test sample. Rinse the sample with anhydrous ethanol more than three times.
2) Prepare the 5% microsphere solution using anhydrous ethanol as solvent. Then, measure the particle size distribution at room temperature and calculate the average particle size as the data at the initial state.
3) Prepare the 5% microsphere solution using the simulated formation water as solvent. Then, put it into the thermostat and record the time as the initial state. Set the temperature of the thermostat to 65 °C. Measure the particle size distribution and calculate the average particle size at 1, 3, 5, 10, and 30 days.

##### SEM and Light Microscope Experiment.

We carried out the SEM experiment and light microscope experiment to find out the morphology and particle size distribution of PMs. As these two experiments can represent the PMs as pictures, we were able to observe the microscopic morphology of PMs and measure the particle size from the image. The experimental procedures are summarized as follows:

1) SEM experiment
  a. After immersing the cover glass in the washing solution for 12 h, rinse it repeatedly with tap water and deionized water and put it in the ultra-clean working platform to dry naturally.
  b. Absorb the microsphere dispersion system with a clean dropper, put one or two drops of the solution on the cover glass. Then, dry it naturally in the ultra-clean working platform to obtain a dry film of the polymer micro-sphere dispersion system.
  c. Fix the dry film of the dispersion system on a glass slide. Take an observation of a wide area and select typical samples with a light microscope.
  d. Fix the selected dry film of the polymer microsphere dispersion system on the template. Spray gold on the surface to make the sample conductive to avoid charge accumulation. After drying, put it into the SEM sample chamber, which should be preheated for 30 min. Pump the sample Vacuum and cool with liquid nitrogen for 30 min. We could observe the sample and select a specific region to take pictures.
2) Light microscope
  a. Stir the microsphere dispersion system evenly.
  b. Put a small amount of microsphere dispersion system on a glass slide with a glass rod and dye with methylene blue.
  c. Place the glass slide on the microscope stage for observation. Then, set the microscope magnification to 400 times, and select an appropriate area to observe and take pictures.

### Experiment Results

#### Hydration Swelling Experiment Results

**FIGURE 2:**
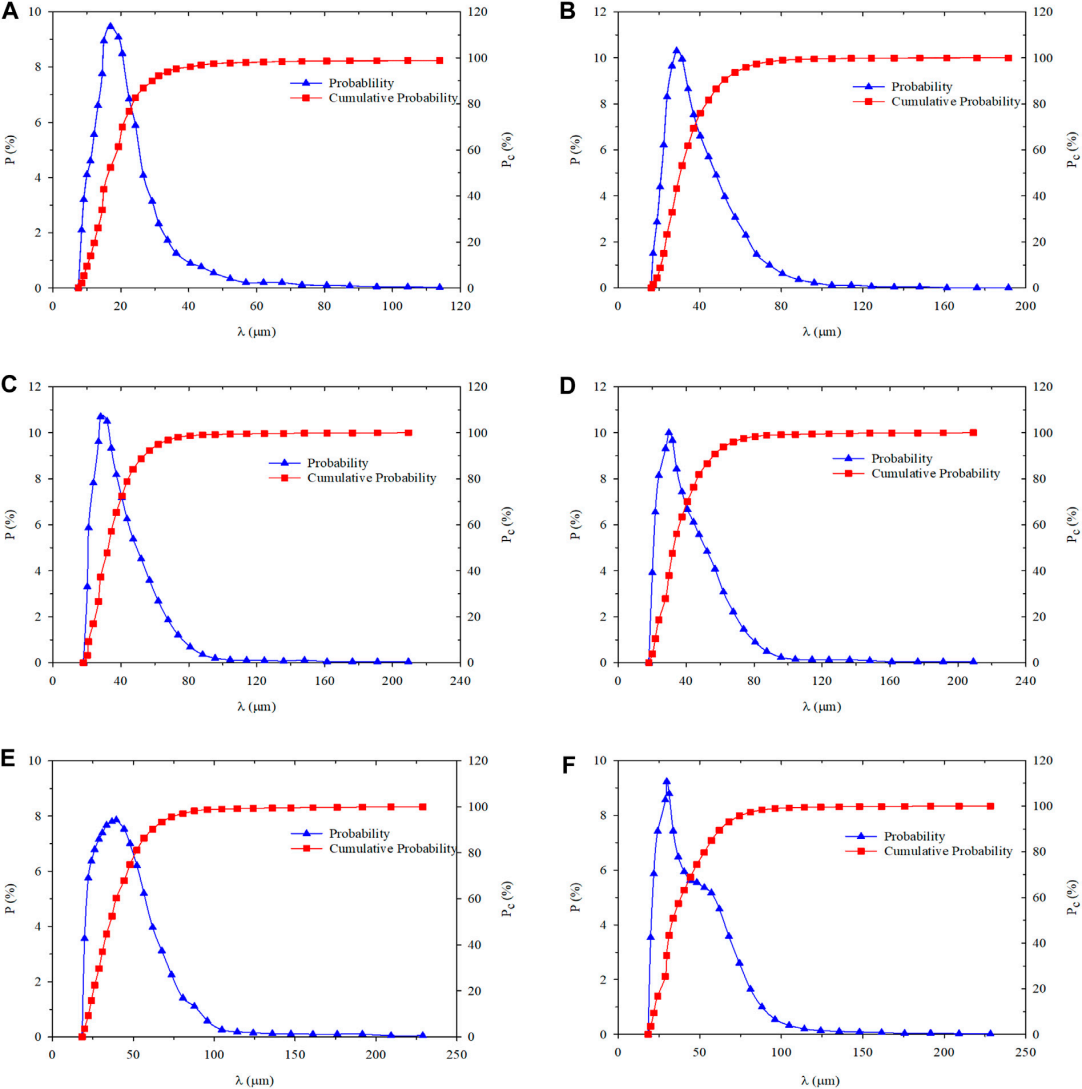
Size probability and the cumulative probability distribution of PMs versus different hydration time: **(A)** Hydration initial state; **(B)** Hydration time 1 day; **(C)** Hydration time 3 days; **(D)** Hydration time 5 days; **(E)** Hydration time 10 days; **(F)** Hydration time 30 days.

The particle size distribution of PMs dispersion system measured in the swelling hydration experiment at 1, 3, 5, 10, and 30 days are shown in Figure 2 and Table 1 (Li, 2015).

**TABLE 1:** The diameter of PMs at different hydration times.

Hydration time/day	λave/μm	*Std/f*
0	18.12	30.25
1	25.31	45.47
3	34.8	55.12
5	36.37	55.83
10	39.35	60.35
30	39.81	60.51

The PMs expanse in the process of hydration can be observed, which leads to the increase of average particle size (*λ*_*ave*_). Its standard deviations (*S*_*td*_) are calculated based on the particle size distribution and the standard deviation increase with the expansion of PMs can be established. The growth of standard deviation indicates that the PMs dispersion system becomes more and more discrete, which means the particle size of PMs is more and more heterogeneous in the same sample.

#### SEM and Light Microscope Experiment Results

**FIGURE 3:**
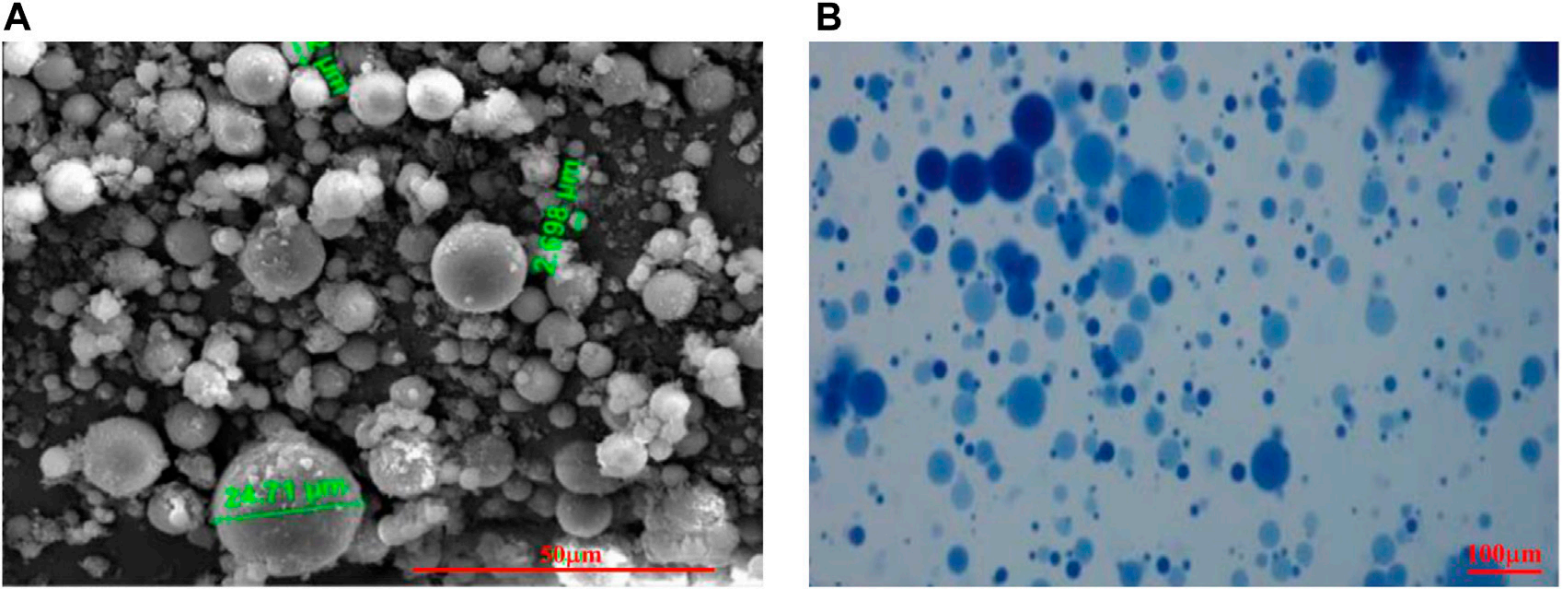
Shape of PMs at different hydration times: **(A)** is the shape of PMs taken with SEM initial state; **(B)** is the shape after hydration 1 day taken with lighting microscope.

**FIGURE 4:**
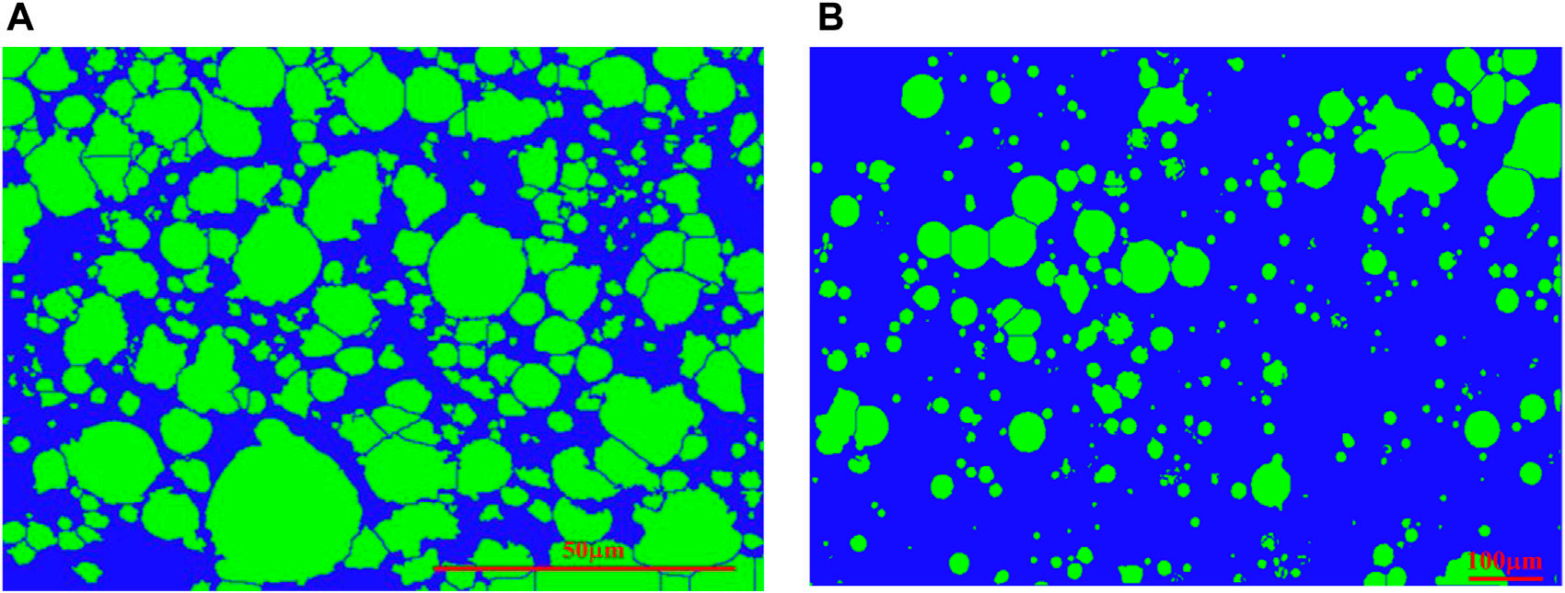
PMs distribution at different hydration times after processing with ImageJ: **(A)** Hydration initial state; **(B)** Hydration time 1 day.

The images of PMs dispersion system are taken with SEM and light microscopes separately at the initial state and 1 day (Li, 2015). Figure 3 shows the shape of PMs. The originally obtained images should be processed by de-hoisting, cropping, and threshold segmentation with ImageJ to count the number of PMs (Amandine et al., 2019; Stolze et al., 2019; Andrialovanirina et al., 2020; Peterson et al., 2020). After converting to grayscale with ImageJ, as shown in Figure 4, import grayscale image into Fractalyse software to calculate the fractal dimension.

### Polymer Microspheres Fractal Dimension Calculation

#### Polymer Microspheres Fractal Dimension Calculation by Cumulative Probability Model

**FIGURE 5:**
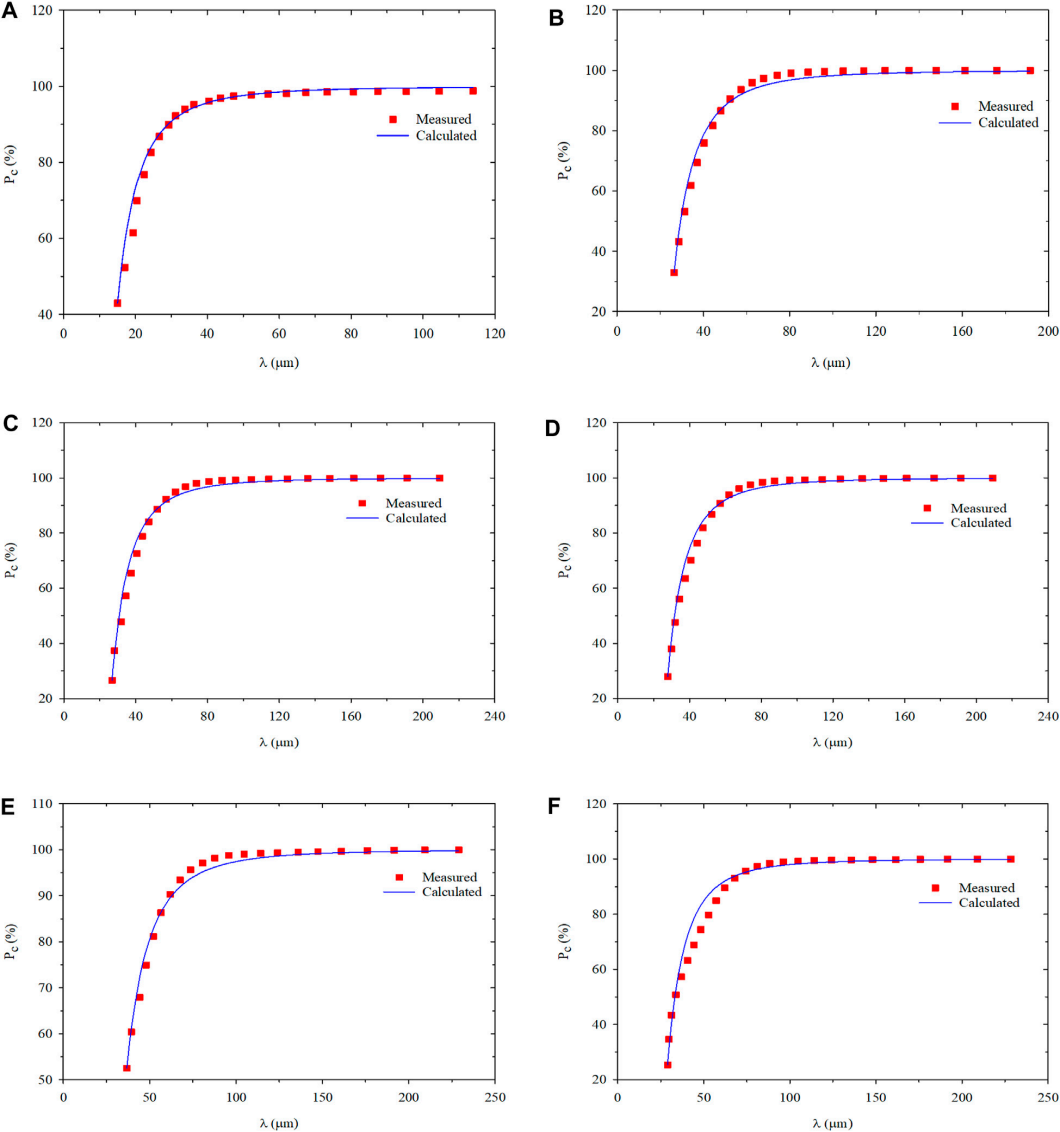
Comparison of size distribution with cumulative probability method of PMs at the different hydration time: **(A)** Hydration initial state; **(B)** Hydration time 1 day; **(C)** Hydration time 3 days; **(D)** Hydration time 5 days; **(E)** Hydration time 10 days; **(F)** Hydration time 30 days.

The fractal dimensions were calculated by fitting the size distribution of PMs measured in the swelling hydration experiment at differents hydrations times. Figure 5 shows the fitting results.

**FIGURE 6:**
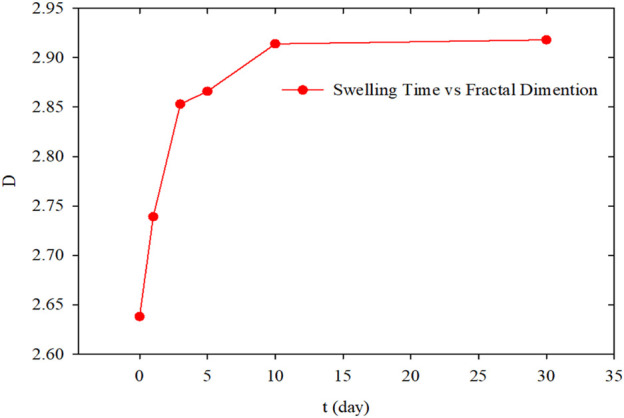
Fractal dimensions calculated with fractal cumulative probability method versus hydration time.

Based on the results shown in Table 2, the relationship between fractal dimensions of PMs dispersion system and hydration time was plotted, as shown in Figure 6. According to the curve, during the preliminary hydration stage, the fractal dimension increases from the initial state to 3 days from the initial state to the same dimension increases from 2.638 to 2.853. After 10 days, the increasing rate of fractal dimension reduces, and fractal dimension barely changes. From the initial state to the end of the swelling hydration experiment, the fractal dimension increases from 2.638 to 2.914. It can be considered a good result with high correlation coefficients between 0.992 and 0.997.

**TABLE 2:** The PMs fractal dimensions D and fitted parameters at different hydration times.

Hydration time/day	λcut/μm	D/f	R2/f
0	15	2.638	0.992
1	26.4	2.739	0.996
3	26.81	2.853	0.993
5	27.84	2.866	0.995
10	36.77	2.914	0.996
30	28.9	2.918	0.997

In the fitting process, the cut value is essential to obtain the best accurate fractal dimension. The variation of this value could change the acceptable value of the fractal dimension. When calculating the cumulative probability with different cut values at the same fractal dimension, the error calculating equation can be expressed as follows:

$$E_{D}=\left|1-{\left(\frac{\lambda_{ocut}}{\lambda_{ocut}}\right)}^{D}\right|\times 100$$ (10)

Where *E*_*D*_ is percentage error *λ*_*ocut*_ is another cut value that is different from the real cut value (*λ*_*cut*_) obtained from the given method, and it could be greater than or less than the real cut value.

From Eq. 8, it can be found that the percentage error highly depends on the fractal dimension and the degree that the cut value differs from the actual cut value. When the fractal dimension is 2.638, and the cut value is 1.05 times the real cut value, the percentage error is 13.73%. However, if the fractal dimension change to 2.918, the percentage error increase to 15.30%. The calculation results indicate that the percentage error increases in the rise of the fractal dimension and the degree that the cut value differs from the actual cut value. Therefore, researchers can not modify the cut value substantially in the process of data fitting.

#### Polymer Microspheres Fractal Dimension Calculation by Box Counting Model

**FIGURE 7:**
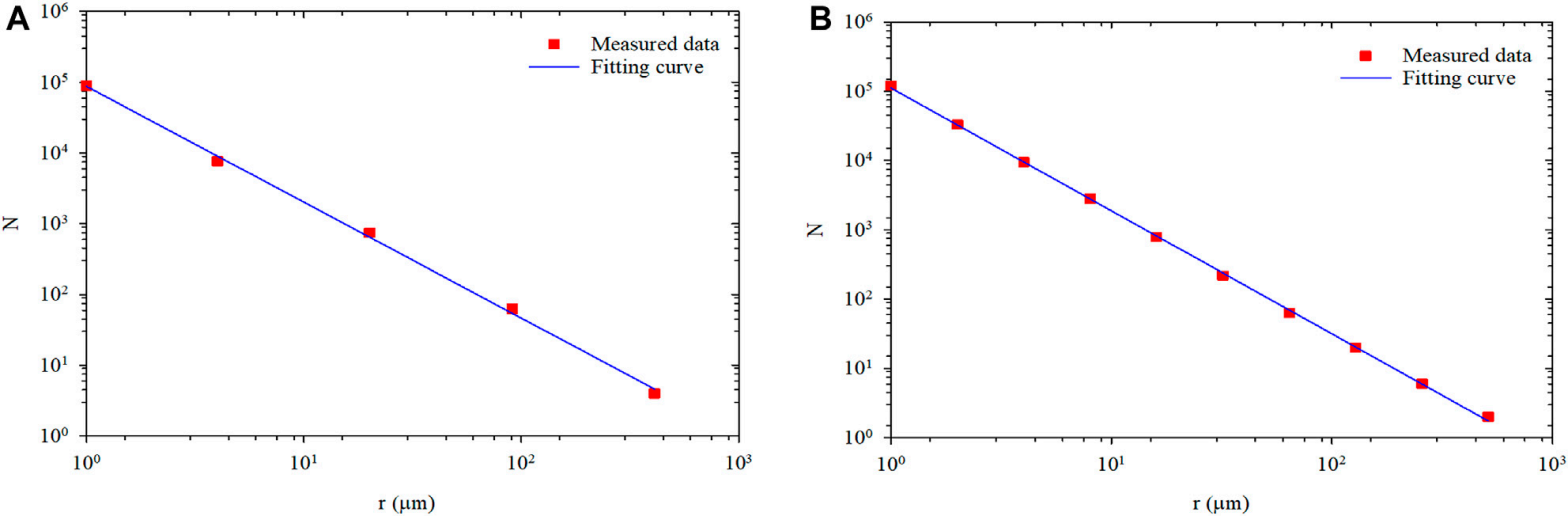
The number of boxes in various box sizes at the different hydration times in log-log coordinate: **(A)** Hydration initial state; **(B)** Hydration 1 day.

The logarithmic plot is the number of boxes in various box sizes obtained from Fractalyse, as shown in Figure 7. The results follow a linear relationship on the logarithmic scale and confirm the statistical fractal nature of the PMs dispersion system. The slope of the two straight lines is 1.638 and 1.741, and the correlation coefficient of the fitted lines is 0.998 and 0.995. Therefore, the fractal dimension for the 3D PMs (*D*_*b3*_) is 2.638 and 2.741.

The fractal dimension calculated results of the PMs dispersion system with the box-counting method and cumulative probability model are shown in Table 3. The results indicate that the calculated value of fractal dimension has a small gap for the same PMs. It also verifies that the cumulative probability model is correct to calculate the fractal dimension of the PMs dispersion system.

**TABLE 3:** PMs fractal dimensions calculated by cumulative probability model and box-counting method.

Hydration time/day	Db2/f	Db3/f	D/f
0	1.638	2.638	2.638
1	1.741	2.741	2.739

## Polymer Microspheres Fractal Dimension Characteristic Analysis

### Space Occupancy Ability

**FIGURE 8:**
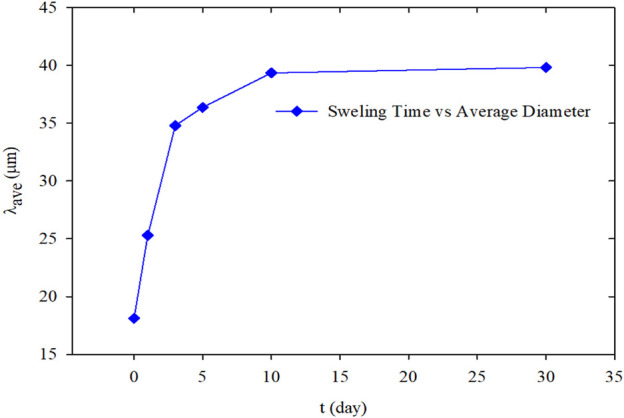
The average size of PMs versus hydration time.

**FIGURE 9:**
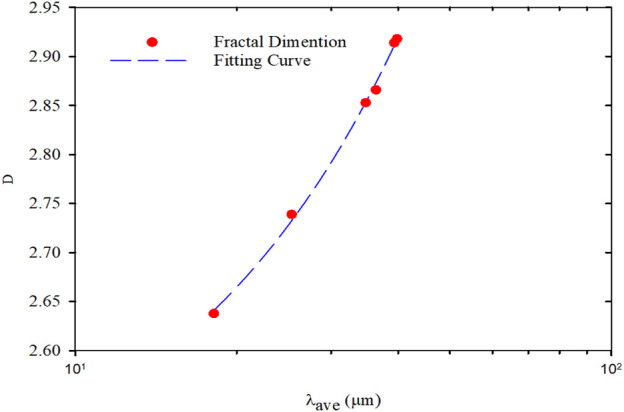
Fractal dimension versus the average size of PMs.

The relationship between the average diameters of PMs and hydration time was plotted based on the hydration swelling experiment results, as shown in Figure 8. According to the curve, the particle diameter of the PMs increased rapidly at the initial stage of hydration. The increase rate decreased gradually, and finally, the particle size barely varied after achieving hydration equilibrium. During the preliminary hydration stage from the initial state to 3 days, the average size of PMs expanded from 18.12 to 34.8 μm. From 3 to 10 days, the increasing rate of the average particle diameter reduced, and the average particle diameter only increases by 4.55 μm. After day 10, the average particle diameter barely varied. Therefore, the system reached balance on the 10th day according to the changing particle size pattern. From the initial state to the hydration equilibrium, the average particle diameter increased from 18.12 to 39.35 μm. The changing of fractal dimension with the average size of PMs were shown in Figure 9. It indicated that the fractal dimension was correlated to the averaged particle size in the Cartesian coordinates. Fractal dimension was linear increase with the average particle size, and the regression correlation coefficient was 0.998. The regression equation can be expressed as follows:

$$D=0.0127\lambda_{ave}+2.4109$$ (11)

The fractal dimension reflects and represents the space occupancy ability of the PMs dispersion system. Furthermore, the larger the PMs average particle size is, the larger the fractal dimension is, and the stronger the ability to occupy space. At the initial hydration stage of PMs, the space-occupying rate quickly increases with the PMs size rapidly increasing, and so does the fractal dimension. When the swelling equilibrium is reached, the particle size of PMs reaches the largest. Meanwhile, the space-occupying rate of the PMs dispersion system reaches the maximum, and so does the fractal dimension. After that, the fractal dimension is barely varied because the space occupancy rate is unchanged when the particle size of PMs ceases to increase.

### Space Occupancy Effectiveness

**FIGURE 10:**
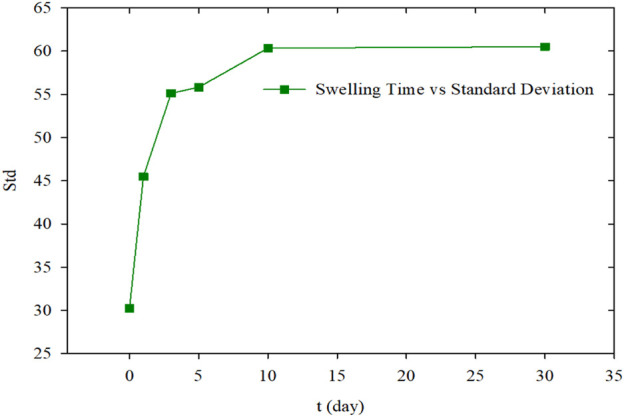
The average size of PMs with different hydration times.

The relationship between the standard deviations of the PMs’ average size and hydration time was shown in Figure 10. It indicated that the standard deviation increased rapidly at the initial hydration stage, but after achieving hydration equilibrium, they barely varied.

**FIGURE 11:**
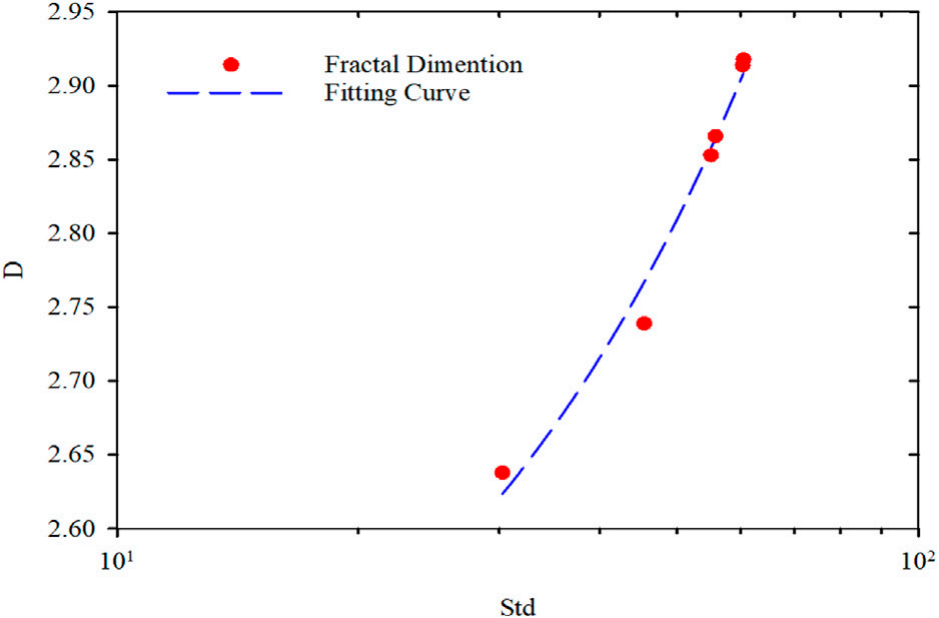
Fractal dimension versus the average size of PMs.

The relationship between fractal dimension and the standard deviations of the PMs’ average size was shown in Figure 11. As the expected deviation increases, the fractal dimension increases. The fractal dimension was correlated to the standard deviation in the semi-logarithmic coordinates. Fractal dimension was linear increase with the standard deviation, and the regression correlation coefficient was 0.998. The regression equation can be expressed as follows:

D=0.0094std+2.338 (12)

The standard deviation of the PMs’ average size represents the space occupancy effectiveness of the PMs dispersion system. In a real reservoir, the size of the pore and throat is a discrete distribution. Therefore, the higher the dispersion of the PMs average particle size, the greater the probability of occupying the reservoir space. As the hydration swells, the particle size distribution of PMs becomes more dispersive. The standard deviation of PMs’ average size increases, and the fractal dimension tends to do so. This change is that the fractal dimension is a measure of the irregularity of the complex system. The more eccentric the system is, the larger the fractal dimension can be. That means the fractal dimension will be larger if the size of the PMs differs considerably. The continuity of the particle size distribution is relatively poor as the fractal dimension represents the uniformity of particle size composition.

## Conclusion

This paper established a new model to calculate the fractal dimension of PMs. Hydration swelling experiments, SEM experiments, and light microscope experiments were carried out to verify this model. Then, the new model and the box counting model were used to calculate the fractal dimension of PMs based on the results of the experiment. The correctness of the new model is verified by comparing the calculation results of the two models. Finally, according to the calculation results of the new model, the fractal dimension characteristics of PMs are analyzed. The major conclusions that can be drawn from this study are as follows:

1) Based on the self-similarity theory, a fractal dimension calculation model that is particular for PMs was established. This model successfully correlates the cumulative probability of the PMs dispersed system with the fractal dimension and makes fractal dimension calculation of PMs more accurately and conveniently.
2) The fractal dimension of PMs calculated by the new model and the box counting model are all 2.638 at the initial state and 2.739 and 2.741 at hydration time as 1 day based on the experiment results. Comparing the calculation results of the two models indicates that the new model can be used to calculate the fractal dimension of PMs. In addition, the hydration swelling results indicate that the PMs reach the hydration equilibrium after 10 days of hydration. Meanwhile, the fractal dimension of PMs increases from 2.638 to 2.918 based on the new model calculation results.
3) The fractal dimension of PMs calculated by the new model indicates that the fractal dimension is linear correlated to the average particle size of PMs and the standard deviation average particle size during the hydration process. Specifically, with the increase of fractal dimension, the average particle size of PMs increases from 18.12 to 39.35 μm, and the standard deviation of average particle size increases from 30.25 to 60.51 μm. That means that the fractal dimension of PMs represents the space occupancy ability and space occupancy effectiveness.

## Data Availability

The original contributions presented in the study are included in the article/Supplementary Material, further inquiries can be directed to the corresponding author.
